# Temperature Distribution of Selected Body Surfaces in Scoliosis Based on Static Infrared Thermography

**DOI:** 10.3390/ijerph17238913

**Published:** 2020-11-30

**Authors:** Anna Lubkowska, Ewa Gajewska

**Affiliations:** 1 Chair and Department of Functional Diagnostics and Physical Medicine, Faculty of Health Sciences, Pomeranian Medical University in Szczecin, 54 Żołnierska Str, 71-210 Szczecin, Poland; 2 Department of Developmental Neurology, Poznan University of Medical Sciences, 49 Przybyszewskiego Str, 60-355 Poznan, Poland; ewagajewska@ump.edu.pl

**Keywords:** scoliosis, thermography, skin temperature, asymmetry

## Abstract

The purpose of the research was to assess the usefulness of thermography as a complementary method in musculoskeletal dysfunction, with particular emphasis on scoliosis. The children, aged 7–16, were classified into one of two groups: the study group—children with scoliosis (*n* = 20), and the reference group—healthy children (*n* = 20). All children underwent anthropometric tests, body mass index determination, four pictures each with a FLIR T1030sc HD thermal imaging camera, and measurement of spinal rotation with a scoliometer (Gima, Italy). There is a temperature differential (about 4 °C) within the upper and lower body in children. In healthy children, differences in temperature of contralateral areas of the body do not exceed 0.5 °C. Thermography is a useful and noninvasive method of assessing muscular tension disbalance in the course of scoliosis. In the case of scoliosis, the areas of the body with a significant thermal asymmetry of the surface are the upper back, thighs, and back of the lower legs. Due to the high positive correlation of the spinal rotation angle with the amount of thermal asymmetry, the areas that should be subjected to a detailed thermal assessment in the supplementary diagnosis of scoliosis using thermovision are the upper back, chest, thighs, and back of the lower legs.

## 1. Introduction

One of the features reflecting the correct body structure and posture is the thermal symmetry of the body surface, i.e., skin temperature, while asymmetry may be a symptom of disorders with a varied background. Many authors have attempted to develop a thermal equation to predict the minimum allowable temperature asymmetry difference above which pathology might be suspected. Depending on the source, this value ranges from 0.3 °C to 0.8 °C [1,2,3]. One of the first studies in this area, published by Uematsu, emphasized that in healthy people the difference in skin temperature between the sides of the body is only 0.24 ± 0.073 °C [4]. In 2012, Vardasca et al. determined precisely that the thermal lateral differentiation in healthy people is 0.4 ± 0.3 °C when the entire body surface is considered, and 0.4 ± 0.15 °C for its individual regions [5]. As a result of lesions, especially inflammatory ones, the areas affected are characterized by increased tissue temperature, which results indirectly in the skin temperature of a given region of the body. Hildebrandt et al. claim that in the case of pathophysiological processes in the body, the difference between symmetrical areas of the body may even reach 1 °C [6]. Due to the sensitivity of the method, one of the main areas of application of thermal imaging is the comparative assessment of the symmetry/asymmetry of the temperature distribution of selected areas of the body. For athletes, both in static and dynamic thermography, especially in response to symmetrical or asymmetrical physical effort and muscle forces, the temperature of the skin areas noted above for selected muscle groups is assessed [7,8,9,10,11,12]. This method is also proposed to determine the effects of the myofascial trigger points treatment [13]. Morphological asymmetry, apart from the arrangement of internal organs, is expressed by the difference in the weight of the right and left side of the body, the length and circumference of the limbs, and the location of the paired parts of the body. Functional asymmetry is related to the difference in muscle strength and range of motion in the joints, which results in the dominance of activities performed with the right or left limbs, in or gait asymmetry [14,15,16]. Symptoms of asymmetry increase with age, especially after four years, when juvenile idiopathic scoliosis can be diagnosed, and if they occur in an uncontrolled manner, symptoms may lead to severe overload changes within the musculoskeletal system, initially functional and later also structural ones. As far as possible diagnostically, they should be prevented from deepening and not always treated as a manifestation of individual variation [17]. Idiopathic scoliosis (IS) is a most common pediatric musculoskeletal disorder with many dysfunctions in the locomotor system, which in our opinion could be the basis for thermal asymmetry, such as improper posture patterns caused by prolonged proprioceptive stimulation in a scoliotic body position; reduction in muscle flexibility; asymmetrical loading of the buttocks, lower limbs, and feet; disturbed gait pattern; reduced mobility of the ribs on the concave side of the curvature of the spine, and abnormal effort of the chest muscles during breathing (the so-called breathing with convexities); these changes are always accompanied by an imbalance in the muscle tone on the side of the body (the so-called neuromuscular imbalance) [5,18,19,20].

Several methods can be used to screen for IS through an initial general surface measurement and a subsequent selected clinical expert evaluation to eventually reach a final radiographic examination, in which the deformity can be detected early and treated to avoid progression [21]. Patients with IS are exposed to many radiographic examinations of their spine throughout the clinical follow-up using the Cobb angle to measure the magnitude of scoliotic curvature, which leads to excessive exposure to harmful radiation. Therefore, it seems justified to conduct research on the use of noninvasive methods as an aid to early screening. The results published on scoliosis research refer to many diagnostic and therapeutic issues, but there are still few studies on the possibility of applying and assessing the usefulness of the thermal imaging method in this disease.

In their research, Vutan et al. [22] and Kwok et al. [23] proposed using thermal imaging among the methods for the diagnosis of scoliosis, as a useful and noninvasive tool.

When undertaking the planning and implementation of this research, it was assumed, based on isolated literature reports, that the muscle tension asymmetry in children with scoliosis may be accompanied by asymmetry in the distribution of body surface temperature, particularly in the front part of the chest, back, and unevenly loaded lower limbs. Therefore, the aim of the study was to assess the diagnostic usefulness of thermography as a complementary method in musculoskeletal dysfunction, with particular emphasis on scoliosis. It was achieved by assessing the symmetry of temperature distribution in selected areas of the trunk and lower limbs in children with scoliosis; comparing the temperature distribution in children with scoliosis to healthy children; and searching for the relationship between the angle of trunk rotation (ATR) and potential thermal asymmetry.

## 2. Materials and Methods

### 2.1. Subjects

The study group consisted of a total of 40 children, both sexes, aged 7–16 years. The research was conducted in accordance with the Helsinki Declaration of the World Medical Association Assembly, and was approved on December 10, 2012 by the Local Ethics Committee of the Pomeranian Medical University, No. KB-0012/151/12. All parents of the children included in this study signed the informed consent form before the beginning of the study. Guidelines in the Strengthening the Reporting of Observational Studies in Epidemiology (STROBE) for case-control studies were applied. The selection of the subjects was deliberate; the children for the study group were recruited from groups qualified for rehabilitation due to scoliosis by a rehabilitation specialist, pediatrician, and/or neurologist in the Rehabilitation Department of the hospital in Choszczno, Poland. The research was conducted with the consent of the management of the health institutions. The control group consisted of healthy children, volunteers, without postural defects. Healthy children were selected from among volunteers from the same town, reporting in response to information disseminated by pediatric care specialists about the possibility of participating in the research. The children were classified into one of two groups: the study group—children with scoliosis (*n* = 20), and the reference group—healthy children (*n* = 20). The inclusion criteria for the study, regardless of the group, were consent of the parent/legal guardian to participate in the research, age of the examined child between 7 and 18 years, and ability to communicate with the child necessary to conduct the examination (the child wanted to undress, meet the examination requirements). Additionally, general good health without metabolic, cardiovascular, neuromuscular, inflammatory, autoimmune, or rheumatic diseases was confirmed by clinical examination. The inclusion criterion for the study group was the presence of scoliosis, as confirmed in a clinical examination by the referring physician (according to the Scoliosis Research Society (SRS); a Cobb angle above 10°). In all children, body height and weight were measured using a mechanical column scale (Seca 711/220) with a stadiometer and the BMI (body mass index) was calculated. The angle of trunk rotation (ATR) was measured in all children with a scoliometer (Gima, Italy) during the Adam’s forward bending [24]. The scoliometer measures the hump appearing as a consequence of the Adam’s test; it is an evaluation tool that has proven highly useful. The scoliometer measures the angle of trunk inclination (ATI or ATR, angle of trunk rotation) and has a high interobserver reproducibility. Scoliometer measurements showed good correlation with the Cobb angle (the gold standard measurement) [25].

### 2.2. Thermographic Measurements

Subsequently, all children were subjected to four thermal imaging scans in anatomical position in anterior–posterior (A/P) projections: frontal plane front upper body, frontal plane front lower body, frontal plane back upper body, and frontal plane back lower body. All thermograms were recorded in digital form. Each of the thermograms taken was subjected to detailed analysis using FLIR TOOLS software, which enabled the determination of specific symmetrical areas of the body on thermograms, which were the basis for assessing the symmetry of temperature distribution. The thermograms show a line along the vertical axis of the body, dividing the anterior and posterior surfaces of the chest and the back into the right (R) and left (L) sides. Subsequently, the following areas of the body were selected for detailed analysis: upper back right (UBR) and left (UBL), lower back right (LBR) and left (LBL), chest right (ChR) and left (ChL), abdominal right (AbR) and left (AbL); lower extremities right and left, taking into account the following areas: tight front right and left (TF R/L), thigh back right and left (TB R/L), shank front right and left (SF R/L), shank back right and left (SB R/L). A FLIR T1030sc HD camera with a detector resolution of 1024 × 768 (786,432 pixels) and thermal sensitivity <0.02 °C was used for the examinations. The recorded parameters were the minimum temperature (T_min_), the maximum temperature (T_max_) and the mean temperature (T_mean_) inside the selected body areas. The average temperature in a given body area, marked as T_mean_, was used to analyze the examination results. The examinations were performed in accordance with the standards of the European Association of Thermology, under thermal comfort conditions after 10 min of acclimation [26,27]. The subjects were positioned so that the optical axis of the lens was normal to the frontal plane, thus ensuring the optimal measurement angle. The skin emissivity was assumed at the level of 0.98. The environmental factors such as light and temperature were kept constant for normalization. The camera was placed onto a tripod. Thermograms were taken in a room with a humidity of 50% and a temperature of 23 ± 1 °C, from a distance of 1.5 m, which meets the criteria for thermal imaging tests. All evaluations were carried out by the same examiner.

### 2.3. Statistical Analysis

Sample size calculation was carried out for comparing the two groups with a t-test for independent samples, using the power analysis test as a function available in STATISTICA (data analysis software system) StatSoft, Inc. (USA 2014); version 12. StatSoft Poland. In pilot study (*n* = 12), the mean ± SD temperature from all of the examined areas was 31.9 ± 1.7 °C for the healthy children (*n* = 6) and 32.7 ± 1.6 °C for those with scoliosis (*n* = 6). A two-tailed hypothesis, an effect size of 0.4, statistical power of a test of 0.8, and α of 0.05 were applied for sample size calculation procedure. The required sample size was calculated as 36 participants. Finally, 40 participants were divided into two groups, healthy and those with scoliosis, with each group containing 20 children.

The normality of the data distribution was verified with the Shapiro–Wilk test. Student’s t parametric test was used to compare the mean values of temperatures from the analyzed body areas, due to the normal distribution. Correlations between the values of the surface temperatures of selected body areas were estimated by calculating the Pearson correlation coefficient. The level of statistical significance adopted was *p* < 0.05.

## 3. Results

Mean values, standard deviation, minimum and maximum values for body height, body weight, and BMI of children and adolescents from the study and reference groups are summarized in Table 1. The study group consisted of children with scoliosis, including 12 girls and 8 boys. The 20-person reference group (10 girls and 10 boys) did not differ significantly from the study group in terms of age. Additionally, when comparing the body height and weight as well as the BMI values within the same sex, no statistically significant differences were found between the subjects, both girls and boys, in relation to these anthropometric features.

**Figure 1:**
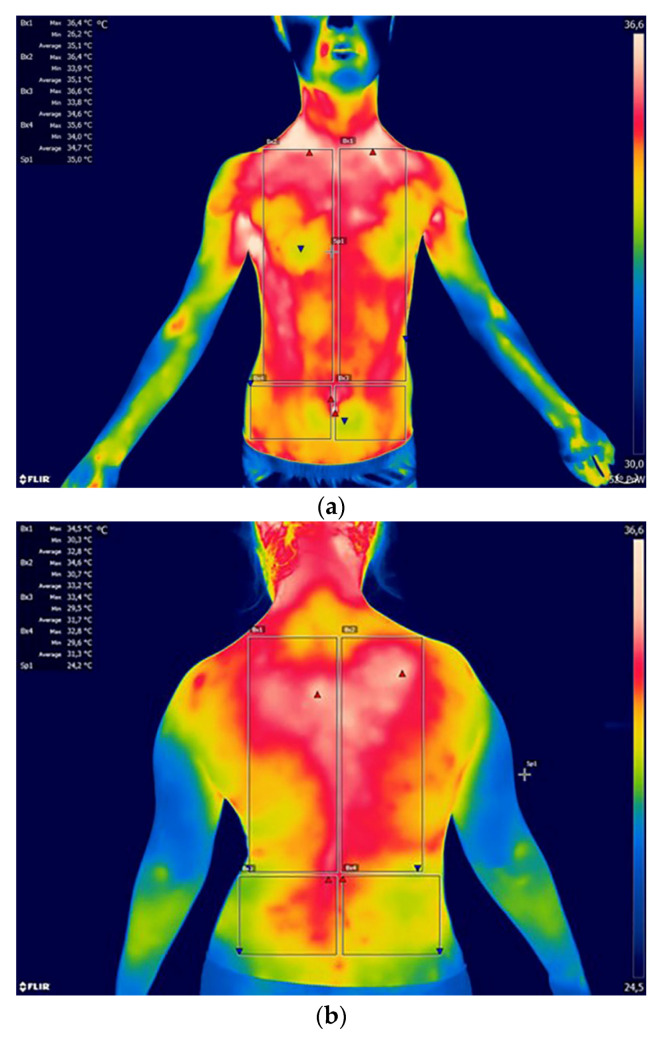
(**a**) Example of a thermogram with marked areas of the chest and abdomen: Bx1—chest left side; Bx2—chest right side; Bx3—abdomen left side; Bx4—abdomen right side. (**b**) Example of a thermogram with marked areas of the back: BX1—upper back left; Bx2—upper back right; BX3—lower back left; BX4—lower back right.

**Figure 2:**
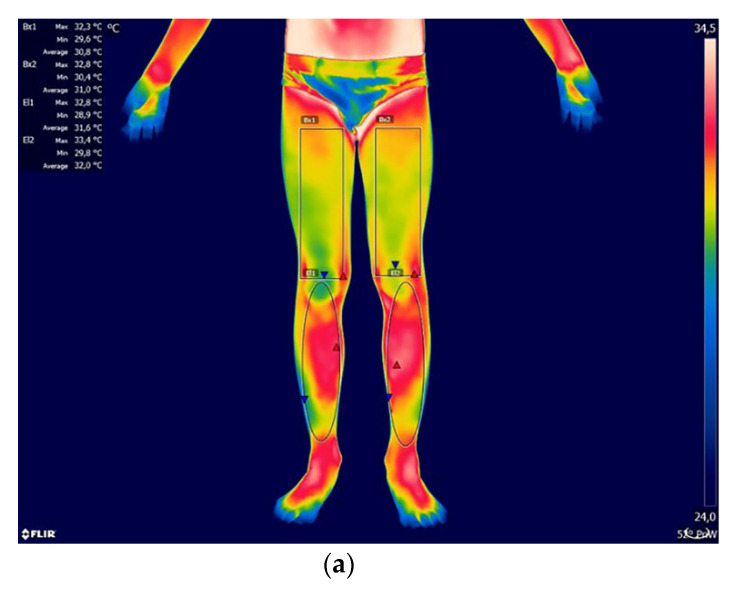

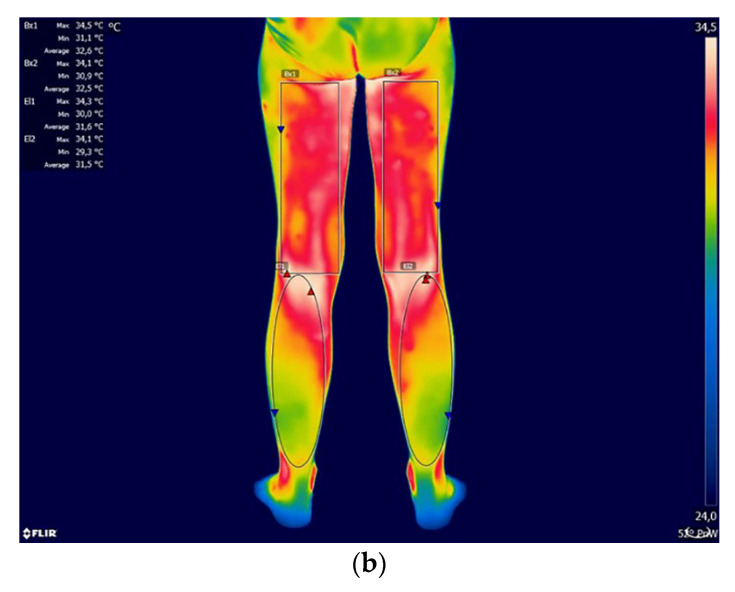
(**a**) Example thermogram with marked areas of the lower limbs (front): Bx1—tight right; Bx2—tight left; El1—shank right; El2—shank left. (**b**) Example thermogram with marked areas of the lower limbs (back): Bx1—thigh left Bx2—thigh right; El1—shank left; El2—shank right.

Almost all girls from the study group were characterized by right-sided, single-arch scoliosis in the thoracic spine. In two of the girls examined, scoliosis also affected the thoracolumbar (Th/L) transition zone in addition to the thoracic (Th) region. Only one of the girls developed left-sided scoliosis in the lumbar (L) region. The mean ATR value in girls, measured with a scoliometer, was 18 ± 9°. The lowest recorded value was 10° and the highest 38°. Left-sided scoliosis (6 subjects) dominated among boys, including two in the lumbar (L) region and four in the thoracolumbar (Th/L) transition zone. The remaining two boys had, similar to the girls, right-sided scoliosis in the thoracic (Th) spine. The mean value of ATR in boys was 17 ± 6°, with the lowest value 8° and the highest 25°. Figure 1a,b and Figure 2a,b show the areas selected for thermographic analysis.

The areas in the A/P plane were analyzed, taking into account the symmetrical division into the right and left sides. When designating areas in FLIR TOOLS software, each time efforts were made to cover the largest possible area on the selected side of the body. Technical limitations of the software related to the possibility of using only circles/ellipses, rectangles or line segments, determined the final selected areas. Table 2 shows the mean values with standard deviation as well as the minimum and maximum temperatures from the analyzed areas of the body for both groups. Significance of differences was shown for the temperatures of the areas of shank back between the studied group of boys and girls. In the group of healthy children, there were no differences between the sexes for the temperatures for all analyzed areas. According to the thermal gradient, from the warmest to the coolest, the areas of the body can be systematized as follows for individual groups:

Studied group

UB = Ch = Ab → LB → → TF = TB = SF = SB

Control group

UB → ChF → LB → Ab → TF = TB = SF = SB

where:

UB—upper back; Ch—chest; Ab—abdominal; LB—lower back; TF—thigh front; TB—thigh back;
SF—shank front; SB—shank back
= no significant difference between areas
→ significant difference between the areas at *p* < 0.05
→→ significant difference between the areas at *p* < 0.01

In most cases, a significant positive correlation was found between the temperature of the analyzed areas, especially in the areas of the upper and lower back. It is noteworthy that in healthy children this correlation was usually stronger (Table 3 and Table 4).

Subsequently, in order to estimate the thermal symmetry of the body surface of the subjects, the difference between the mean temperatures of the given area on the right and left side (∆T_R/L_) was calculated for each subject and for each area. In the analysis, using the Shapiro–Wilk test, the normality of the distribution of results of the thermal analyses was confirmed; therefore, in order to determine the significance of differences between the groups with scoliosis and the reference group, Student’s t-test was used for variables independent of the groups. The differences in temperature between the sides of the body were compared for the given areas, both in terms of sex and combined for the whole groups, carrying out the analyses according to the following scheme: girls with scoliosis vs. girls from the reference group, boys with scoliosis vs. boys from the reference group, and all subjects with scoliosis vs. all subjects from the reference group. The results of the mean values of the arithmetic differences for individual areas and the significance of differences are summarized in Table 5, for the study and reference groups, respectively, broken down by sex. Table 6 shows the results for the entire study group of girls and boys with scoliosis and for the entire reference group.

When analyzing the thermal asymmetry in children with scoliosis within the assessed areas, it can be observed that it was marked in the areas of the upper back (∆ T_R/L_ = 0.4 ± 0.1 °C for both girls and boys), front and back thighs (∆T_R/L_ = 0.4 ± 0.1 °C and 0.3 ± 0.1 °C in girls and ∆T_R/L_ = 0.5 ± 0.1 °C and 0.4 ± 0.1 °C in boys), but it was most strongly expressed on back shanks, where the contralateral temperature differences were ∆T_R/L_ = 0.5 ± 0.2 °C in girls and 0.5 ± 0.1 °C in boys. Considering the whole study group without division into sex, these differences were similar for these areas, i.e., for upper back (∆T_R/L_ = 0.4 ± 0.1 °C), front and back thighs (∆T_R/L_ = 0.4 ± 0.1 °C and ∆T_R/L_ = 0.3 ± 0.1 °C, respectively), and for back shanks (∆T_R/L_ = 0.5 ± 0.2 °C) (Table 5 and Table 6).

Another element of the research was to estimate the relationship between the value of ATR and the temperature difference of the same body areas on the right and left side. A strong correlation was demonstrated between this variable and the ∆T_R/L_ values for the upper back (r = 0.624; *p* < 0.05) and a very strong correlation with the chest temperature (r = 0.857; *p* < 0.05).

## 4. Discussion

The main purpose of the research presented was to assess the diagnostic usefulness of thermography and evaluate the temperature distribution according to thermal symmetry in children with scoliosis. Thermal imaging is widely used for the comparative assessment of the temperature distribution symmetry of selected body areas, both in static and dynamic tests. The use of thermal imaging to assess the symmetry of the muscular effort involved in the stabilization of posture while walking with asymmetric load was described by Awrejcewicz et al. [28]. They describe which muscle groups play the greatest part in postural stabilization and compensate for the additional load, and which are minimally responsible for it, indicating that asymmetric weight has the greatest impact on the thigh on the loaded side, and as a result of asymmetric loading, the temperature difference increased due to its compensation. The temperature difference in the thigh area increased from 0.1 to 1.3 °C. These authors stated clearly that thermal imaging supports the assessment of the stage of spine degeneration, including scoliosis. By measuring the temperature on the body surface, we indirectly assess the blood supply to the tissues lying under the measurement area. Many factors disturb the circulation in the skin, the main ones being inflammation, ischaemia, and sympathetic dysfunction. Owing to infrared thermography, we can indirectly suspect the presence of pathology in thermally changed areas. Thermography can be a useful tool in diagnosing and assessing dysfunctions and injuries within the muscles, but it should always be remembered that individual variation of the distribution of body surface isotherms requires reference not only to healthy people but also to the contralateral side of the subject. Such an assumption was made when starting the described research. When planning the research, the aim was to assess the temperature distribution of selected body areas in children with scoliosis in comparison to healthy children, primarily with regard to the thermal symmetry of the right and left sides of the body. For this purpose, the temperature differences between the right and left sides of the body were calculated for each subject and for each area separately. Based on the knowledge so far, it was assumed that the asymmetry persisted in the degree of muscle stretch, flexibility, tension, and activity, which may cause differences in surface temperatures over specific muscle groups. This goal was achieved based on the baseline study related to the assessment of the distribution of body surface temperatures in children. In the literature, there are only single reports describing the temperature values of the areas analyzed by the authors of this research, and in order to meet the standardization conditions for thermal imaging tests and the reliability of the results and comparative analyses, it seems absolutely necessary.

The analysis of the results obtained showed that the areas of the upper body parts (chest, abdomen, back) are much warmer (by about 4 °C) than the lower parts (thigh, shank) both in healthy children and those with scoliosis. The warmest area in both study groups was the upper back and the chest. In children with scoliosis, a significant temperature gradient occurred successively from the abdominal area to the lower part of the back, and significantly lower temperatures were recorded in the area of the lower limbs, i.e., thighs and shanks. It can be observed that in healthy children, the abdominal area was cooler compared to the lower part of the back, which may indicate greater abdominal overload in children with scoliosis. This would confirm the conclusions of the study by Awrejcewicz et al. cited previously. Comparing the values of the temperatures obtained in this research with the data from the literature, it can be concluded that the area-related thermal differentiation is comparable in terms of the value and direction of the temperature gradient between the upper and lower body parts. In their study, Dębiec-Bąk et al. showed the highest temperatures for the body surface of the upper back (32.5–32.9 °C) and the chest (32.3–32.7 °C), and the lowest ones for the lower limbs (28.5–29.1 °C). At the same time, it should be noted that the authors of the cited study did not differentiate the area of the lower limbs into the thighs and the shanks [29]. Referring to the cited studies, it can be stated that although the measurement conditions seem to be comparable in terms of thermal comfort (based on the comparison of the measurement methodology), the temperature values obtained in this research and the cited studies differ from each other. This is confirmed not only by the large individual variation of body surface temperatures, but above all by the need to relate the results obtained to the temperature values recorded with a specific model of a thermal imaging camera, in repeatable measurement conditions, and, most importantly, by contralateral comparisons in individual subjects as the most important reference point. In our opinion, this is the most sensitive point of thermal imaging tests, which should be borne in mind when operating in this research area and, at the same time, is easily eliminated at the stage of research planning. We should also mention the earlier two-stage study by Dyszkiewicz et al. [30], the initial results of which were controversial and confirmed the usefulness of thermal imaging for the assessment of muscle asymmetry only in selected cases of scoliosis, e.g., “mirror image” thoracolumbar scoliosis. In a study published later by these authors [31], the values of body surface temperatures in children with scoliosis were very similar to those obtained in this research: 33–35 °C in the upper back and 31.5–32.5 °C in its lower parts. These authors indicated that even under physiological conditions, the difference between the upper and lower back can be up to 4.3 °C. They explain the occurrence of such thermal differentiation of the analyzed areas by greater involvement of the paraspinal muscles stabilizing the thoracic spine as compared to the lumbar one. Additionally, when assessing the thermal symmetry of the back, they found that as the bending of the spinal curvature according to the Gruca’s classification increased from the first to the second degree, the thermal asymmetry of the concave side to the convex one worsened, from a difference being on average 0.8 °C to 1.2 °C. Following the detailed studies, they confirmed clearly the usefulness of thermography to assess the asymmetry of muscle activity, especially in the case of idiopathic scoliosis. Referring to the results of Dyszkiewicz and Kuna [31], it can be stated that the difference in temperature between the upper and lower part of the back observed in this research, amounting to approximately 2 °C in healthy children and only 1 °C in the group with scoliosis, may confirm the imbalance of muscle tensions that determine stabilization of the spine in relation to physiological conditions.

When analyzing the thermal asymmetry in children with scoliosis within the assessed areas, it can be observed that it was marked in the areas of upper back and front and back thighs, but it was most strongly expressed on back shank. The difference in the temperature distribution confirming the occurrence of thermal asymmetry occurred both in comparisons between groups, taking into account the division by sex and in comparisons jointly for all children without gender division.

This confirmed the hypothesis that in people with scoliosis, muscle tension asymmetry and general muscle imbalance may be accompanied by asymmetry in the distribution of body surface temperature. Moreover, areas where it is clearly marked were selected. The relationship between curvature size and thermal asymmetry, described in the literature, is also confirmed in this research, which showed a correlation between the value of the angle of trunk rotation and the temperature difference between the right and left sides of the body. Interestingly, there was only a correlation with the temperature difference in the chest and upper back areas, but nevertheless it was very strongly expressed. It is noteworthy that while for the back area it coincides with its strong thermal asymmetry compared to healthy children, no statistically significant thermal asymmetry was found for the chest area in children with scoliosis when compared to healthy children. On the other hand, it was the chest area that turned out to be the one in which temperature was higher in children with scoliosis, placing it in the hierarchy of thermal gradients comparable to the upper back, which was not observed in healthy children. This seems to be a particularly important observation from the study, with significant practical potential in two aspects. First, therapeutic measures should take into account possible effect on the muscle groups of these parts of the body. Secondly, it can be postulated that the areas that should be subjected to a detailed thermal assessment in terms of their potential asymmetry in the complementary or screening diagnosis of scoliosis with the use of thermal imaging are the upper part of the back, the chest, and additionally the thigh and the back of the shanks. Due to the proven effectiveness of conservative treatment in the form of specific physiotherapy or corset treatment, early detection and nonsurgical intervention of idiopathic scoliosis reduces the number of cases that require surgery [32].

A key point to be considered in the assessment of idiopathic scoliosis is screening. Screening diagnosis of scoliosis cannot be based on a radiological assessment due to both the cost and invasiveness of the examination as well as organizational limitations. At present, the proposed method for screening tests is the assessment of the angle of trunk rotation using a trunk forward bending test (Adams test) and scoliometer [33], but at the same time it is postulated that this test has a higher referral rate and less precision when it is the only means of assessment compared with that of other screening methods that use more than one form of screening [34]. Coehlo et al. [25] showed that the correlation between the scoliometer measurements and radiograph analyses was good (r = 0.7, *p* < 0.05). The confirmation of the occurrence of thermal asymmetry, especially in specific areas of the body surface in children with scoliosis, indicates the usefulness of the thermal imaging method as an additional method for scoliosis screening. IR thermography can detect significant differences between the paraspinal muscles on the left and right sides of the body, including differences in the thoracolumbar region where scoliotic conditions are commonly found. It is worth noting that the mean value of the angle of trunk rotation in the children from the study group varied, averaging 18 ± 9° (in the range from 10° to 38°), which, based on the analysis of correlation, leads to the conclusion that the thermal asymmetry of selected body areas increases with the increase in the value of this indicator, which becomes another argument for further research on the use of thermal imaging in the diagnosis of scoliosis.

Despite the very promising results, we are aware of the limitations of research. The results presented, due to the small number of subjects and diversity of spinal deformities, can be considered preliminary; increasing the number of research participants seems to be of particular importance. It also seems necessary to extend the search for relationships between thermography results and the parameters of radiological analyses (especially with the value of Cobb angle) in order to establish the accuracy, sensitivity, and reliability of this method.

## 5. Conclusions

The proposed method in this article establishes some of the groundwork for using IR thermography in detecting the signs of scoliosis. Based on this research, it can be concluded that in children there is a characteristic thermal differentiation of the surfaces of the upper and lower parts of the body. In healthy children, the differences in the temperature of the identical areas of the right and left side of the body proves the occurrence of thermal symmetry, while in the case of scoliosis, there is thermal asymmetry, mainly in the areas of the upper back, thigh, and back shank. Thermography is a useful and noninvasive method, and due to the high positive correlations of the angle of trunk rotation with the size of thermal asymmetry, the areas that should be subjected to a detailed thermal assessment in complementary diagnosis of scoliosis using thermography are the upper back, chest, thighs, and back shanks. Thermography may be considered as a method that supports scoliosis screening, but continued research into its reliability and accuracy is needed.

## Figures and Tables

**Table 1:** Anthropometric characteristics of the subjects from particular groups.

	Study Group				Control Group			
	♀		♂		♀		♂	
	X	Min	X	Min	X	Min	X	Min
	±SD	Max	±SD	Max	±SD	Max	±SD	Max
Age	12	7	9.7	7	11.3	7	10.9	6
[years]	± 2.5	16	± 3.3	15	± 2.9	15	± 2.8	15
Body mass [kg]	42.2	20	33	24	46	38	43.3	22
	± 11.6	63	±9.9	48	± 12.5	68	± 19.9	73
Body height [m]	1.5	1.3	1.4	1.3	1.5	1.4	1.6	1.4
	± 0.1	1.7	±0.1	1.7	± 0.1	1.6	±0.2	1.8
BMI [kg/m^2^]	18.2	12.2	15.7	13.5	20	13.6	16.9	11.6
	± 3.7	23.6	± 1.8	18.9	± 3.3	24.9	± 3.4	21.4

**Table 2:** Mean, minimum, and maximum values for the temperatures of selected body areas in girls and boys with scoliosis, taking into account the differences between the sexes within the groups.

	♀ Study Group		♀ Control Group		♂ Study Group		♂ Control Group	
	X	Min	X	Min	X	Min	X	Min
	± SD	Max	± SD	Max	±SD	Max	± SD	Max
	[°C]	[°C]	[°C]	[°C]	[°C]	[°C]	[°C]	[°C]
UBR	34.6	32.9	34.3	33.9	35.1	34.8	34.6	33.5
	±1.0	36.1	±0.4	35.5	±0.3	35.7	±0.9	36.2
UBL	34.2	32.6	34.2	33.8	34.8	34.3	34.7	33.5
	±1.0	35.8	±0.5	35.5	±0.4	35.6	±0.8	36
LBR	33.4	31.7	32.9	32.2	34	33	35.8	32
	±1.2	35	±0.8	34.8	±0.6	34.8	±1.2	35.7
LBL	33.4	31.5	32.8	32.1	33.7	32.8	33.5	31.8
	±1.3	35.9	±0.8	34.7	±0.6	34.5	±1.2	35.6
ChR	34.6	32.9	33.6	32.9	34.6	34.4	34.2	33
	±0.9	35.7	±0.7	35.5	±0.2	35	±0.9	35.5
ChL	34.6	32.9	33.7	32.9	34.7	34.5	34.2	33
	±1.0	35.7	±0.7	35.5	±0.2	35.1	±0.9	35.5
AbR	34	31.7	32.7	31.5	34.1	33.6	33.7	32.2
	±1.2	35.4	±1.2	35.7	±0.5	34.6	±1.3	35.7
AbL	34	31.9	32.6	31.4	34.1	33.6	33.7	32.3
	±1.1	35.5	±1.3	35.7	±0.5	34.6	±1.3	35.7
TFR	30.6	28.4	29.9	27.6	30.7	30.1	30.2	28.3
	±1.3	33.4	±1.6	32.3	±0.4	31.2	±1.6	32.3
TFL	30.4	28.1	29.9	27.7	31.2	30.8	30.1	27.7
	±1.5	33	±1.6	32.5	±0.3	31.5	±1.9	32.5
TBR	30.9	29.1	30.2	28	31.6	31.1	30.8	28.5
	±1.3	32.9	±1.7	33.5	±0.3	32	±2.1	33.5
TBL	30.9	29.4	30.3	27.9	31.7	31.7	30.8	28.6
	±1.3	33.4	±1.7	33.3	±0.1	31.8	±2.0	33.3
SFR	30.9	29.4	30.9	28.6	31.6	31.4	31.1	29.8
	±1	32.4	±1.4	32.8	±0.2	31.9	±1.3	32.8
SFL	30.9	29.3	30.6	28.5	31.5	30.9	31.1	29.6
	±1.1	32.4	±1.4	33.1	±0.4	32	±1.4	33.1
SBR	30.3	28.6	30.1	27.7	31.5	30.9	30.3	28.3
	±0.9 *	31.6	±1.6	32.4	±0.6	32.5	±1,7	32.4
SBL	30.6	29	30	27.9	31.5	30.4	30.3	28.2
	±0.9 *	31.9	±1.5	32.4	±0.5	31.9	±1.7	32.4

* significance of sex differences in the groups of children with scoliosis at *p* < 0.01; UB R/L—upper back right/left side; LB R/L—lower back right/left side; Ch R/L—chest right/left side; Ab R/L—abdominal right/left side; TF R/L—thin front right/left; TB R/L—thin back right/left; SF R/L—shank front right/left; SB R/L—shank back right/left.

**Table 3:** Values of the correlation coefficient between the mean values of the temperatures of selected body areas in children with scoliosis.

	UBL	UBR	LBR	LBL	ChR	ChL	AbR	AbL	TFR	TFL	TBR	TBL	SFR	SFL	SBR	SBL
UBL	-	**0.97**	**0.82**	**0.76**	**0.72**	**0.76**	**0.65**	**0.62**	0.16	0.3	0.27	0.34	0.45	0.39	0.35	0.45
UBR	**0.97**	-	**0.82**	**0.77**	**0.75**	**0.79**	**0.7**	**0.67**	0.2	0.33	0.26	0.37	0.47	0.42	0.33	0.45
LBR	**0.82**	**0.82**	-	**0.96**	**0.76**	**0.78**	**0.67**	**0.65**	**0.44**	**0.54**	**0.55**	**0.64**	**0.49**	0.48	0.57	0.74
LBL	**0.76**	**0.77**	**0.96**	-	**0.77**	**0.76**	**0.67**	**0.66**	**0.5**	**0.54**	**0.53**	**0.64**	**0.47**	**0.45**	**0.49**	**0.67**
ChR	**0.72**	**0.75**	**0.76**	**0.77**	-	**0.98**	**0.92**	**0.87**	**0.47**	**0.46**	0.42	**0.49**	**0.44**	0.38	0.24	**0.47**
ChL	**0.76**	**0.79**	**0.78**	**0.76**	**0.98**	-	**0.91**	**0.86**	**0.48**	**0.49**	0.43	**0.5**	**0.53**	**0.46**	0.28	**0.5**
AbR	**0.65**	**0.7**	**0.67**	**0.67**	**0.92**	**0.91**	-	**0.97**	**0.54**	**0.5**	0.37	**0.51**	**0.52**	**0.45**	0.38	**0.5**
AbL	**0.62**	**0.67**	**0.65**	**0.66**	**0.87**	**0.86**	**0.97**	-	**0.57**	**0.55**	0.41	**0.54**	**0.57**	**0.51**	0.42	**0.51**
TFR	0.16	0.2	**0.44**	**0.5**	**0.47**	**0.48**	**0.54**	**0.57**	-	**0.93**	**0.78**	**0.84**	**0.81**	**0.79**	**0.63**	**0.61**
TFL	0.3	0.33	**0.54**	**0.54**	**0.46**	**0.49**	**0.5**	**0.55**	**0.93**	-	**0.89**	**0.89**	**0.86**	**0.85**	**0.74**	**0.76**
TBR	0.27	0.26	**0.55**	**0.53**	0.42	0.43	0.37	0.41	**0.78**	**0.89**	-	**0.92**	**0.68**	**0.67**	**0.7**	**0.756**
TBL	0.34	0.37	**0.64**	**0.64**	**0.49**	**0.5**	**0.51**	**0.54**	**0.84**	**0.89**	**0.92**	-	**0.76**	**0.76**	**0.78**	**0.77**
SFR	**0.45**	**0.47**	**0.49**	**0.47**	**0.44**	**0.53**	**0.52**	**0.57**	**0.81**	**0.86**	**0.68**	**0.76**	-	**0.97**	**0.68**	**0.59**
SFL	0.39	0.42	**0.48**	**0.45**	0.38	**0.46**	**0.45**	**0.51**	**0.79**	**0.85**	**0.67**	**0.76**	**0.97**	-	**0.63**	**0.57**
SBR	0.35	0.33	**0.57**	**0.49**	0.24	0.28	0.38	0.42	**0.63**	**0.74**	**0.7**	**0.78**	**0.68**	**0.63**	-	**0.86**
SBL	**0.45**	**0.45**	**0.74**	**0.67**	**0.47**	**0.5**	**0.5**	**0.51**	**0.61**	**0.76**	**0.75**	**0.77**	**0.59**	**0.57**	**0.86**	-

The Pearson r correlation coefficients marked in bold are significant for *p* < 0.05. UB R/L—upper back right/left side; LB R/L—lower back right/left side; Ch R/L—chest right/left side; Ab R/L—abdominal right/left side; TF R/L—thin front right/left; TB R/L—thin back right/left; SF R/L—shank front right/left; SB R/L—shank back right/left.

**Table 4:** Values of the correlation coefficient between the mean values of the temperatures of selected body areas in healthy children.

	UBL	UBR	LBR	LBL	ChR	ChL	AbR	AbL	TFR	TFL	TBR	TBL	SFR	SFL	SBR	SBL
UBL	-	0.97	**0.92**	**0.96**	**0.9**	**0.89**	**0.83**	**0.83**	0.3	0.25	0.43	0.41	0.4	0.39	0.28	0.25
UBR	**0.97**	**-**	**0.94**	**0.96**	**0.91**	**0.91**	**0.85**	**0.85**	0.38	0.36	**0.521**	**0.5**	**0.46**	**0.46**	0.37	0.34
LBR	**0.92**	**0.94**	**-**	**0.98**	**0.96**	**0.95**	**0.94**	**0.93**	**0.53**	**0.48**	**0.64**	**0.63**	**0.58**	**0.57**	**0.5**	**0.46**
LBL	**0.96**	**0.96**	**0.98**	**-**	**0.94**	**0.93**	**0.9**	**0.89**	**0.44**	0.39	**0.57**	**0.55**	**0.51**	**0.5**	0.41	0.38
ChR	**0.9**	**0.91**	**0.96**	**0.94**	**-**	**0.99**	**0.98**	**0.98**	**0.6**	**0.56**	**0.7**	**0.69**	**0.64**	**0.65**	**0.57**	**0.56**
ChL	**0.89**	**0.91**	**0.95**	**0.93**	**0.99**	**-**	**0.97**	**0.96**	**0.65**	**0.61**	**0.74**	**0.73**	**0.69**	**0.69**	**0.62**	**0.6**
AbR	**0.83**	**0.85**	**0.94**	**0.9**	**0.98**	**0.97**	**-**	**0.99**	**0.68**	**0.65**	**0.7**	**0.76**	**0.69**	**0.71**	**0.65**	**0.64**
AbL	**0.83**	**0.85**	**0.93**	**0.899**	**0.98**	**0.96**	**0.99**	**-**	**0.64**	**0.6**	**0.74**	**0.71**	**0.65**	**0.67**	**0.6**	**0.59**
TFR	0.3	0.38	**0.53**	**0.443**	**0.6**	**0.65**	**0.68**	**0.64**	**-**	**0.98**	**0.97**	**0.97**	**0.95**	**0.96**	**0.99**	**0.98**
TFL	0.25	0.36	**0.48**	**0.39**	**0.56**	**0.61**	**0.65**	**0.6**	**0.98**	**-**	**0.96**	**0.96**	**0.92**	**0.93**	**0.98**	**0.99**
TBR	0.43	**0.52**	**0.64**	**0.57**	**0.7**	**0.74**	**0.79**	**0.74**	**0.97**	**0.96**	**-**	**0.99**	**0.94**	**0.96**	**0.97**	**0.96**
TBL	0.41	**0.5**	**0.63**	**0.55**	**0.69**	**0.73**	**0.76**	**0.71**	**0.97**	**0.96**	**0.99**	**-**	**0.95**	**0.96**	**0.97**	**0.97**
SFR	0.4	**0.46**	**0.58**	**0.51**	**0.64**	**0.69**	**0.69**	**0.65**	**0.95**	**0.92**	**0.94**	**0.95**	**-**	**0.99**	**0.96**	**0.94**
SFL	0.39	**0.46**	**0.57**	**0.5**	**0.65**	**0.69**	**0.71**	**0.67**	**0.96**	**0.93**	**0.96**	**0.96**	**0.99**	**-**	**0.96**	**0.95**
SBR	0.28	0.37	**0.5**	0.41	**0.57**	**0.62**	**0.65**	**0.6**	**0.99**	**0.98**	**0.97**	**0.97**	**0.96**	**0.96**	**-**	**0.99**
SBL	0.25	0.34	**0.46**	0.38	**0.56**	**0.6**	**0.64**	**0.59**	**0.98**	**0.99**	**0.96**	**0.97**	**0.94**	**0.95**	**0.99**	**-**

The Pearson r correlation coefficients marked in bold are significant for *p* < 0.05. UB R/L—upper back right/left side; LB R/L—lower back right/left side; Ch R/L—chest right/left side; Ab R/L—abdominal right/left side; TF R/L—thin front right/left; TB R/L—thin back right/left; SF R/L—shank front right/left; SB R/L—shank back right/left.

**Table 5:** Differences in the values of mean temperature of selected areas between the right and left side of the body (∆T_R/L_) listed separately for girls and boys in both groups.

	∆T_R/L_			
	♀ Study Group	♀ Control Group	♂ Study Group	♂ Control Group
	X± SD [°C]		X ± SD [°C]	
UBR	0.4 ± 0.1	0.0 ± 0.1 ***	0.4 ± 0.1	0.1 ± 0.1 ***
UBL				
LBR	0.2 ± 0.2	0.1 ± 0.1	0.2 ± 0.1	0.1 ± 0.1 *
LBL				
ChR	0.1 ± 0.14	0.1 ± 0.11	0.1 ± 0.1	0.02 ± 0.0 **
ChL				
AbR	0.2 ± 0.1	0.1 ± 0.1	0.025 ± 0.0	0.1 ± 0.1 **
AbL				
TFR	0.4 ± 0.1	0.19 ± 0.1 **	0.5 ± 0.1	0.3 ± 0.2
TFL				
TBR	0.3 ± 0.1	0.15 ± 0.1 ***	0.4 ± 0.1	0.1 ± 0.1 ***
TBL				
SFR	0.2 ± 0.1	0.16 ± 0.1	0.22 ± 0.1	0.2 ± 0.1
SFL				
SBR	0.5 ± 0.2	0.22 ± 0.1 **	0.5 ± 0.1	0.1 ± 0.1 ***
SBL				

Significance level of intergroup differences within sex: * *p* < 0.05, ** *p* < 0.01, *** *p* < 0.001. UB R/L—upper back right/left side; LB R/L—lower back right/left side; Ch R/L—chest right/left side; Ab R/L—abdominal right/left side; TF R/L—thin front right/left; TB R/L—thin back right/left; SF R/L—shank front right/left; SB R/L—shank back right/left.

**Table 6:** Differences in the values of mean temperature of selected areas between the right and left side of the body (∆T_R/L_) combined for girls and boys from the study group and the reference group.

	∆T_R/L_	
	♀ + ♂ Study Group	♀ + ♂ Control Group
	N = 20	N = 20
	X ± SD [°C]	X ± SD [°C]
UBR	0.4 ± 0.1 ***	0.1 ± 0.1
UBL		
LBR	0.2 ± 0.2	0.1 ± 0.1
LBL		
ChR	0.1 ± 0.1	0.0 ± 0.1
ChL		
AbR	0.1 ± 0.1	0.1 ± 0.1
AbL		
TFR	0.4 ± 0.1 **	0.2 ± 0.1
TFL		
TBR	0.3 ± 0.1 **	0.1 ± 0.1
TBL		
SFR	0.2 ± 0.1	0.2 ± 0.1
SFL		
SBR	0.5 ± 0.2 ***	0.2 ± 0.1
SBL		

Significance between study and control group of children** *p* < 0.01, *** *p* < 0.001. UB R/L—upper back right/left side; LB R/L—lower back right/left side; Ch R/L—chest right/left side; Ab R/L—abdominal right/left side; TF R/L—thin front right/left; TB R/L—thin back right/left; SF R/L—shank front right/left; SB R/L—shank back right/left.
